# Supplementary material for: Head-Out Water-Based Protocols to Assess Cardiorespiratory Fitness—Systematic Review
Source: Int J Environ Res Public Health. 2020 Oct 2;17(19):7215. doi: 10.3390/ijerph17197215 (PMC7579306; doi:10.3390/ijerph17197215)
Supplement: Supplementary file 1 [file ijerph-17-07215-s001.pdf]

International Journal of
Environmental Research and Public Health

MDPI

1 **Table S1.** Example of search parameters.

Search details for PubMed:
((("oxygen consumption"[MeSH Terms] OR ("oxygen"[All Fields] AND "consumption"[All Fields]) OR "oxygen consumption"[All Fields]) OR (("oxygen"[MeSH Terms] OR "oxygen"[All Fields]) AND uptake[All Fields]) OR ("cardiorespiratory fitness"[MeSH Terms] OR ("cardiorespiratory"[All Fields] AND "fitness"[All Fields]) OR "cardiorespiratory fitness"[All Fields]) OR (Cardiorespiratory[All Fields] AND response[All Fields]) OR (Aerobic[All Fields] AND capacity[All Fields]) OR (Aerobic[All Fields] AND fitness[All Fields])) AND (Aqua[All Fields] OR Aquatic[All Fields] OR Water-based[All Fields])) AND (("exercise"[MeSH Terms] OR "exercise"[All Fields]) OR Testing[All Fields] OR ("research design"[MeSH Terms] OR ("research"[All Fields] AND "design"[All Fields]) OR "research design"[All Fields] OR "test"[All Fields]) OR Protocol[All Fields])

2

3

4 **Table 2.** Quality assessment of included studies.

**Author, Year**	**Study Purpose**	**Literature**	**Design**	**Sample**		**Outcomes**		**Protocol**			**Results**				**Conclusions were Appropriate Given Study Methods and Results?**	**Total**
				**Was the Sample Described in Detail?**	**Was Sample Size Justified?**	**Were the Outcomes Reliable?**	**Were the Outcomes Valid?**	**Protocol was Described in Detail?**	**Contamination was Avoided?**	**Cointervention was Avoided?**	**Results were Reported in Terms of Statistical Significance?**	**Were the Analysis Methods Appropriate?**	**Clinical Importance was Reported?**	**Drop Outs were Reported?**		
Alberton et al., 2013 [41]	**1**	**1**	**0**	**1**	0	0	0	1	1	1	1	1	1	1	1	11
Alberton et al., 2013 [42]	1	1	0	1	0	0	1	1	1	1	1	1	1	1	1	12
Alberton et al., 2014 [43]	1	1	0	1	0	0	1	1	1	1	1	1	1	1	1	12
Alberton et al., 2016 [44]	1	1	0	1	1	0	1	1	1	1	1	1	1	1	1	13
Antunes et al., 2015 [45]	1	1	0	1	1	0	1	1	1	1	1	1	1	1	1	13
Bartolomeu et al., 2017 [46]	1	1	0	1	1	0	1	1	0	0	1	1	1	1	1	11
Broman et al., 2006 [33]	1	1	0	1	0	0	1	1	0	0	1	1	0	1	1	9
Brown et al., 1997 [26, 27]	1	1	0	1	0	0	1	1	0	0	1	1	1	1	1	10
Brown et al., 1998 [52]	1	1	0	1	0	0	1	1	0	0	1	1	0	1	1	9

Brubaker et al., 2011 [60]	1	1	0	1	0	0	1	1	1	1	1	1	1	1	1	12
Butts et al., 1991 [53]	1	0	0	1	0	0	1	1	0	0	1	1	1	1	1	9
Butts et al., 1991 [54]	1	1	0	1	0	0	1	1	0	0	1	1	1	1	1	10
Campbell et al., 2003 [37]	1	1	0	1	0	0	0	1	0	0	1	1	1	1	1	9
Choi et al., 2015 [61]	1	1	0	1	0	0	1	1	0	0	1	1	1	1	1	10
Colado et al., 2019 [62]	1	1	1	1	1	0	1	1	1	1	1	1	1	1	1	14
Conti et al., 2008 [48]	1	1	0	1	0	0	1	1	0	1	1	1	1	1	1	11
Conti et al., 2015 [49]	1	1	0	0	0	0	1	1	0	0	1	1	1	1	0	8
Costa et al., 2017 [63]	1	1	0	1	0	0	0	1	1	1	1	1	1	1	1	11
D'Acquisto et al., 2015 [29]	1	1	1	1	0	0	1	1	0	0	1	1	1	1	1	11
Dowzer et al., 1999 [47]	1	1	0	1	0	0	1	1	0	1	1	1	1	1	1	11
Fisher et al., 2019 [30]	1	1	0	1	1	0	1	1	0	1	1	1	1	1	1	12
Frangolias et al, 1996 [39]	1	1	0	1	0	0	1	1	1	0	1	1	1	1	1	11
Gayda et al., 2010 [24]	1	1	1	1	0	1	1	1	1	1	1	1	1	1	1	14
Greene et al., 2011 [32]	1	1	0	1	0	0	1	1	1	0	1	1	1	1	1	11
Kanitz et al., 2015 [55]	1	1	0	1	1	0	1	1	1	1	1	1	1	1	1	13

Kruel et al., 2013 [50]	1	1	0	1	1	0	1	1	1	1	1	1	0	1	1	12
Melton-Rogers et al., 1996 [56]	1	1	0	1	0	0	1	1	0	0	1	1	1	1	1	10
Mercer et al., 1998 [57]	1	1	0	1	0	0	1	1	0	0	1	1	1	1	1	10
Meredith-Jones et al., 2009 [36]	1	1	0	1	0	1	1	1	0	0	1	1	1	1	1	11
Michaud et al., 1995 [34]	1	1	0	1	0	0	1	1	1	1	1	1	1	1	1	12
Michaud et al., 1995 [40]	1	1	0	1	0	1	1	1	1	1	1	1	1	1	1	13
Nagle et al., 2017 [31]	1	1	1	1	0	1	0	1	0	0	1	1	1	0	1	10
Ogonowska-Slodownik et al., 2019 [58]	1	1	1	1	0	1	1	1	1	1	1	1	1	1	1	14
Phillips et al., 2008 [35]	1	1	0	1	0	0	1	1	0	0	1	1	1	1	1	10
Pinto et al., 2016 [64]	1	1	0	1	0	0	1	1	0	1	1	1	1	1	1	11
Schaal et al., 2012 [38]	1	1	0	1	0	0	1	1	0	1	1	1	1	1	1	11
Silvers et al., 2007 [65]	1	1	0	1	0	0	1	1	1	0	1	1	1	1	1	11
Silvers et al., 2008 [66]	1	1	0	1	0	1	0	1	0	0	1	1	1	1	1	10

Svedenhag et al., 1992 [59]	1	1	0	1	0	0	1	1	0	0	1	1	0	1	1	9
Town et al., 1991 [36]	1	1	0	0	0	0	1	1	1	0	1	1	1	1	1	10
Yazigi et al., 2013 [51]	1	1	0	1	0	0	1	1	1	1	1	1	1	1	1	12
